# 797 The Development and Management of Neck Burn Scar Contracture Recurrence: A Single-Center Retrospective Cohort Study

**DOI:** 10.1093/jbcr/irae036.338

**Published:** 2024-04-17

**Authors:** Jose Antonio Arellano, Tiffany Jeong, Mario Alessandri-Bonetti, Sumaarg Pandya, Hilary Liu, Guy M Stofman, Francesco Egro

**Affiliations:** University of Pittsburgh Medical Center, Pittsburgh, PA; University of Pittsburgh Medical Center, Pittsburgh, PA; University of Pittsburgh Medical Center, Pittsburgh, PA; University of Pittsburgh Medical Center, Pittsburgh, PA; University of Pittsburgh Medical Center, Pittsburgh, PA; University of Pittsburgh Medical Center, Pittsburgh, PA; University of Pittsburgh Medical Center, Pittsburgh, PA

## Abstract

**Introduction:**

The neck has the highest incidence and poorest burn scar contraction outcomes, due to the region being thin and mobile. After burn injury, the neck was determined to be the area with the most limited range of motion due to wound contractures. While there has been some exploration of various surgical techniques, there is a paucity of literature regarding long-term outcomes based on different surgical management strategies. The aim of this study was to evaluate the long-term outcomes of the treatment of neck scar contractures and evaluate surgical strategies according to their long-term effectiveness in avoiding contracture recurrence (CR).

**Methods:**

A retrospective study was conducted to review outcomes of neck contracture release after burn injury. All patients operated between April 2009 and December 2022 at a single institution were included.

**Results:**

During the period from 2009 to February 2023, 51 patients with a mean age of 32.9±20.3 years received treatment for neck burns by plastic surgery. Of these 51 patients, 39.2% (n=20), developed a neck burn scar contracture after their acute burn treatment and were included in this study. The burn injuries were most commonly thermal (n=19, 95%). All burn injuries were full thickness burns, with an average neck defect size of 130.5±106.0 cm2. Overall, 45 surgical scar release procedures were performed on the 20 patients who developed a neck contracture. Patients underwent 3.4±2.3 surgeries on average. While 25% of patients only received one surgery to treat neck contracture, some patients underwent as many as 8 surgeries. Contracture recurrence (CR) was the most common complication and occurred in 28.9% of the cases. The mean TBSA did not significantly differ in CR patients (26.7±14.9%) and no CR patients (44.5±30.2%). However, there was a significant difference (p=0.01), in the average neck defect size between CR patients (198.5±108.3 cm2) and no CR patients (81.1±75.1 cm2). When following patients post-operatively, on average contracture recurrence (256.4±249.4days) presented later than initial contracture (128.9±84.9 days). We suspect that this is because patients are followed more closely in the acute setting.

**Conclusions:**

TBSA was not associated with CR, suggesting that the defect size of the neck is more relevant than TBSA when considering a patient’s risk for developing CR. Significantly, this current study supports that there is a difference in average neck defect size between patients who develop CR and those who do not.

**Applicability of Research to Practice:**

When taken into consideration with what is known with respect to the development of initial BSC, physicians should be attentive to neck defect size when understanding a patient’s risk for neck BSC sequelae. For patients undergoing contracture release procedures, more frequent follow-up may be warranted to decrease the time to clinical presentation.

